# Nutrient density and affordability of aquatic foods in the FAO uFISH database assessed using Nutrient Rich Food (NRF) indices

**DOI:** 10.3389/fnut.2025.1675142

**Published:** 2025-11-03

**Authors:** Emma Johnsson, Cristen Harris, Adam Drewnowski

**Affiliations:** ^1^Food Systems, Nutrition and Health Program, School of Public Health, University of Washington, Seattle, WA, United States; ^2^Center for Public Health Nutrition, University of Washington, Seattle, WA, United States

**Keywords:** nutrient profiling, nutrient rich food, aquatic foods, fish, shellfish, priority micronutrients, affordability index

## Abstract

**Background:**

Fish and shellfish are valuable sources of high-quality protein, vitamins, and minerals. Their nutrient density and price vary by species.

**Objectives:**

This study aimed to determine nutrient density and nutrient affordability per unit cost of fish and shellfish in the FAO/INFOODS uFISH database.

**Methods:**

Two versions of the Nutrient Rich Food (NRF) index were constructed. The NRF was based on a positive subscore, NRn, and a negative subscore, LIM. The NR6 subscore was the sum of percent daily values (%DV) for 6 priority micronutrients widely identified to be lacking in low and middle-income countries, including iron, zinc, calcium, vitamin A, vitamin B12, and folate. The NR9 subscore, specifically tailored to include micronutrients relevant to fish and shellfish, was the sum of %DV for protein, calcium, iron, potassium, magnesium, selenium, vitamin A, vitamin D, and omega-3 fatty acids. The LIM subscore was based on saturated fat and sodium. In both cases NRFn.2=NRn−LIM , with %DV calculated per 100 g and capped at 100%. Food prices, obtained from GlobeFish, were converted to the cost per 100 g protein. A new Affordability Index for Aquatic Foods was calculated as nutrient density per unit cost.

**Results:**

The NRF6.2 score identified mollusks, and especially bivalves, as the best aquatic source of priority micronutrients. The NRF9.2 revealed small pelagic fish as the most nutrient rich, followed by bivalves. Mackerel, tilapia, squid, and mussels provided the most protein, NRF6.2 nutrients, and NRF9.2 nutrients per penny.

**Conclusion:**

Fish and shellfish are an affordable source of protein, omega-3 fatty acids, and priority micronutrients. However, there were species differences in cost and nutrient density, with mackerel, mussels, tilapia, octopus, and squid scoring the highest for nutrient density at an affordable cost.

## 1 Introduction

Protein-energy malnutrition and micronutrient shortfalls across lower- and middle-income countries (LMIC) continue to challenge global public health (1, 2). Dietary shortfalls of iron, zinc, folate, iodine, selenium, vitamin A, and vitamin B12 among women and young children (1) have been linked to child stunting, anemia, impaired cognitive development, and later-life osteoporosis and heart disease (3). Protein-energy malnutrition is reported to affect nearly 150 million people worldwide (4).

Nutrient-rich aquatic foods have the potential to alleviate protein-energy malnutrition and micronutrient shortfalls. Seafood is a sustainable source of high-quality protein (5) and is particularly rich in iron, calcium, zinc, selenium, iodine, and vitamin B12 (6). Affordability is a critical determinant of diet quality in LMIC, where households spend a larger share of their income on food. In some LMIC, seafood is the main—if not the only—source of animal protein (7), being less costly than either meat or dairy.

Yet, according to FAO sources (7), the potential for aquatic foods to improve LMIC diets remains largely untapped. FAO food balance sheets for 2022 show higher per capita seafood availability in high-income countries at 12.6 kg/capita/year, compared to 5.3 kg/capita/year in the LMIC (7), underscoring a nutritional equity gap where populations with the greatest burden of micronutrient deficiencies have the least available nutrient-dense seafood. Additionally, consumption of aquatic foods in LMIC may be limited by competition with more affordable nutrient-poor alternatives, evolving cultural preferences, environmental vulnerabilities, and weak market and processing infrastructure.

The present goal was to assess the nutrient density and affordable nutrient density of different types of fish and shellfish. Data for the present study came from the FAO/INFOODS Global Food Composition Database for Fish and Shellfish (uFISH) (8). The FAO database for fish and shellfish was aggregated by phylum, subphylum, or by biological distinction (finfish, crustaceans, and mollusks).

Two versions of the Nutrient Rich Food (NRF) index were the measures of nutrient density of fish and shellfish. The NRF was based on nutrients to encourage and nutrients to limit. We also created an Affordability Index for Fish and Shellfish by linking NRF nutrient profiles with available retail prices from GlobeFish (9). Integrating nutrient density with cost opens the door to future assessments of affordable nutrient density, a key theme for the FAO (10) and other international agencies. This study is among the first to assess nutrient density of aquatic foods in the uFISH database and to explore relations between nutrient density and cost.

## 2 Methods

### 2.1 Nutrient composition database

The FAO/INFOODS Global Food Composition Database for Fish and Shellfish (uFISH) (8) was developed to enable countries and researchers to incorporate data on fish and shellfish into food composition tables. The FAO uFISH database integrates multiple FAO databases into a single system. Among the databases are statistical data on capture fisheries, species catalog, and market and trade data from GLOBEFISH. The database was developed following consultation with stakeholders, including FAO member states and regional fishery bodies. The FAO continues to expand uFISH by adding new data sets and improving data validation and quality controls.

The uFISH database provides reliable, globally sourced data for the energy and nutrient content of fish, crustaceans, and mollusks. A total of 515 food items is included, covering 78 unique fish and shellfish species. The data are expressed per 100 grams edible portion of each aquatic food. Available are data on energy (kcal/100 g), macronutrients, including protein and amino acids, fats and fatty acids, including omega-3 fatty acids, and vitamins and minerals. The uFISH database includes some underutilized fish, and codes each item by species, habitat, and capture method. The uFISH database is the most comprehensive resource to date, along with databases from Denmark (11), Japan (12), and the US (13).

The uFISH database obtained from FAO was missing selected values for omega-3 fatty acids, selenium, iodine, potassium, and other nutrients for some aquatic foods. Comparable aquatic foods were first identified in the USDA Food and Nutrient Database for Dietary Studies 2021–2023 and used to fill in missing data, primarily for crabs, crayfish, oysters, mussels, and trout. Any still missing values were obtained from Frida (11), a Danish food database, for crabs, crayfish, lobsters, prawns, oysters, and mussels, if no comparable USDA data were available for those items.

### 2.2 Aggregation by phylum, subphylum and biological distinction

Aquatic foods in the uFISH database were classified by phylum, subphylum, or category. The first distinction was made mollusks (phylum Mollusca), crustaceans (subphylum Crustacea) and finfish. Finfish and mollusks were further organized into 7 broad taxonomic groups. The finfish groups were small pelagic (*n* = 12), cichlid (*n* = 24), demersal (*n* = 96), and salmonid (*n* = 69). The Mollusk groups were bivalve (*n* = 66), gastropod (*n* = 12), and cephalopod (*n* = 36). Aquatic foods were also assigned to 21 categories by species, namely abalone, bass, catfish, clam, cod, conch, crab, crayfish, lobster, mackerel, mussel, octopus, oyster, pike, salmon, scallop, shrimp or prawn, sole, squid, tilapia and trout. The uFISH database also classifies aquatic foods by state (cooked, raw, and preserved), habitat (farmed versus wild), and habitat salinity (freshwater versus saltwater). The relevant groupings are shown in Supplementary Table 1.

### 2.3 Nutrient profiling

The Nutrient Rich Food Index (NRF) contains two subscores: NRn, based on a variable number n of nutrients to encourage, and LIM, normally based on 3 nutrients to limit. The present analysis used 2 nutrients to limit to create the LIM score: sodium and saturated fat. This food group does not contain added sugar, and added sugar data were not available.

The NRF6.2 score was based on 6 priority micronutrients, identified as lacking in diets of LMIC (14), namely iron, zinc, calcium, vitamin A, vitamin B12, and folate. The classic NRF9.2 score was based on protein, fiber, calcium, iron, potassium, magnesium, vitamin A, vitamin C, and vitamin D. Aquatic foods contain negligible amounts of fiber and vitamin C. Hence, a new variant called NRF9.2 was developed to capture the unique nutritional qualities of aquatic foods, which included protein, selenium, calcium, iron, potassium, magnesium, vitamin A, vitamin D, and omega-3 fatty acids. No corrections for protein digestibility were completed as all seafood is a complete source of protein (15).

The NRF scores were calculated as the sum of percent daily values (%DVs) of nutrients to encourage minus the sum of %DVs for saturated fat and sodium (LIM). Percent daily value was calculated per 100 g and capped at 100%. Reference daily values for micronutrients included in the NRF6.2 and NRF9.2 were those used by the US Food and Drug Administration (16), which are designed to reflect the nutrient needs of a general, healthy U.S. population aged 4 years and older (not sex-specific), in alignment with the DRIs from the National Academies. %DV is calculated based on a standard 2,000 calorie diet.

### 2.4 Protein affordability and NRF price indices

Price data came from the FAO Globefish price dashboard and European price report for October 2024 (9). Aquatic food species groups in the uFISH database were matched with similar items in the price report. Prices were converted from Euro to USD using the exchange rate as of October 2024 (1.09) (17). Abalone and crayfish were excluded as no price data was available. Percent yields were obtained from the USDA Agriculture Handbook No. 102 and used to calculate the price per kg of edible portion of fish and shellfish (18). Cost per 100 g protein was calculated by dividing the cost per 100 g by protein content per 100 g. The Affordability Index for Fish and Shellfish was calculated by dividing the mean NRF score by the cost per kg, edible portion.

### 2.5 Statistical analyses

Statistical analysis compared nutrient content of aquatic foods by phylum, habitat, biological, ecological and taxonomic groups. Mean, median, and standard deviations of specific nutrients including protein, amino acids, fatty acids, vitamins, and minerals as well as of NRF6.2 and NRF9.2 were calculated for each aquatic food group. SPSS (Statistical Package for the Social Sciences v 16.0), JMP Pro (v 17 and v 18), and R version 4.4.1 were used for statistical analyses. Tests of significance were based on one-way ANOVA to reveal statistically significant differences between group means. Tukey’s HSD post-hoc tests between paired groups to determine which groups differ significantly from one another for each nutrient or NRF score. The significance level for all statistical tests was *p* < 0.05.

## 3 Results

### 3.1 Nutrient density of finfish, mollusks, and crustaceans

Table 1 shows the mean values (M) and standard deviations (SD) of selected minerals, vitamins, and omega-3 s per 100 g by aquatic food type. The first comparisons were for mollusks, crustaceans and finfish. There were significant differences between groups for all nutrients. Mollusks contained more (*p* < 0.0001), zinc (*p* < 0.0001), magnesium (*p* < 0.0001), and selenium (*p* < 0.0001) than crustaceans or finfish. Mollusks and crustaceans contained similar levels of calcium. Finfish were higher in potassium than mollusks (*p* < 0.0001) and crustaceans (*p* < 0.0001). Crustaceans were higher than finfish in iron (*p* = 0.0006), zinc (*p* = 0.008), magnesium (*p* = 0.002), and selenium (*p* < 0.0001).

**Table 1:** Nutrient content per 100 g and NRF 6.2 and 9.2 scores of crustaceans, finfish, and mollusks and mollusks by group.

Nutrient	Crustacean (*N* = 152)		Finfish (*N* = 249)		Mollusk (*N* = 114)		*p*-value
	Mean	SD	Mean	SD	Mean	SD	
Protein (g)	20.1^b^	3.09	21.5^a^	2.74	19.5^b^	10.0	0.0014
Iron (mg)	1.16^b^	0.89	0.59^c^	0.58	4.38^a^	2.84	<0.0001
Zinc (mg)	2.63^b^	1.45	0.88^c^	1.48	8.06^a^	11.78	<0.0001
Calcium (mg)	98.91^a^	119.43	24.25^b^	18.31	117.82^a^	121.72	<0.0001
Potasssium (mg)	281^b^	84.0	369^a^	71.1	303^b^	132	<0.0001
Magnesium (mg)	44.0^b^	13.6	28.6^c^	4.60	96.0^a^	91.4	<0.0001
Selenium (mcg)	45.93^b^	15.52	29.26^c^	13.11	65.96^a^	33.59	<0.0001
Folate (mg)	20.3^b^	14.1	9.41^c^	3.22	29.6^a^	30.5	<0.0001
Vitamin B12 (mcg)	4.10^b^	2.92	3.60^b^	2.93	16.42^a^	11.89	<0.0001
Vitamin A (mcg)	18.87^b^	18.27	9.44^c^	7.58	41.99^a^	32.41	<0.0001
Vitamin D (mcg)	0.00^b^	0.00	6.89^a^	8.27	0.18^b^	0.38	<0.0001
Omega-3 (g)	0.20^b^	0.10	0.81^a^	0.94	0.63^a^	0.46	<0.0001
NRF6.2	123.76^b^	33.83	85.20^c^	31.19	161.95^a^	55.78	<0.0001
NRF9.2	167.19^b^	27.27	153.01^c^	51.36	214.09^a^	43.92	<0.0001

Mollusks by group							
Nutrient	Bivalve (*N* = 66)		Cephalopod (*N* = 36)		Gastropod (*N* = 12)		*P*-value
	Mean	SD	Mean	SD	Mean	SD	
Protein (g)	13.6^e^	5.74	25.2^b^	6.48	34.8^a^	11.6	<0.0001
Iron (mg)	5.21^a^	3.12	2.88^b^	1.95	4.27^ab^	1.26	<0.0001
Zinc (mg)	10.6^a^	14.9	4.10^b^	2.27	5.76^ab^	3.19	<0.0001
Calcium (mg)	117^b^	73.5	44.5^d^	20.1	344^a^	213	<0.0001
Potasssium (mg)	240^d^	98.5	408^a^	129	334^abc^	92.3	<0.0001
Magnesium (mg)	67.7^b^	31.1	79.5^b^	24.3	301^a^	163	<0.0001
Selenium (mcg)	63.5^a^	37.7	74.1^a^	21.8	27.9^bc^	6.34	<0.0001
Folate (mcg)	40.9^a^	35.8	16.2^bc^	5.72	7.44^bc^	1.08	<0.0001
Vitamin B12 (mcg)	19.8^a^	11.0	11.2^b^	11.6	5.38^bcd^	0.95	<0.0001
Vitamin A (mcg)	55.2^a^	35.7	26.9^b^	14.1	14.6^bcde^	1.29	<0.0001
Vitamin D (mcg)	0.25^e^	0.44	0.09^e^	0.26	0.00^de^	0.00	<0.0001
Omega-3 (g)	0.66^b^	0.39	0.74^b^	0.56	0.12^c^	0.01	<0.0001
NRF6.2	192^a^	50.0	132^b^	24.2	84.8^cd^	16.2	<0.0001
NRF9.2	229^b^	45.6	207^c^	15.2	150^de^	27.8	<0.0001

Means within rows that do not share a superscript letter are significantly different at the *p* < 0.05 level based on Tukey’s HSD post hoc tests.

No seafood category was a good source of folate (<10% DV). Mollusks contained more vitamin A and B12 as compared to finfish and crustaceans (all *p* < 0.0001). Finfish were high in vitamin D (*p* < 0.0001) and contained more omega-3 fatty acids than crustaceans (*p* < 0.0001) and mollusks (*p* = 0.051). Mollusks and crustaceans contained similar amounts of vitamin D, but mollusks were significantly higher in omega-3s compared to crustaceans (*p* < 0.0001).

All seafood categories provided >20%DV of protein, selenium, and vitamin B12 this was reflected in overall NRF nutrient density scores. Mollusks provided >20% DV of omega-3 fatty acids, vitamin B12, iron and zinc. Finfish provided >20% DV of vitamin D and omega-3 s, but were lower in iron and zinc. This was reflected in overall NRF6.2 and NRF9.2 nutrient density scores shown in Table 1. Mollusks had the highest nutrient density scores on both indices, with values significantly above those for crustaceans and finfish on both NRF6.2 (*p* < 0.0001) and NRF9.2 (*p* < 0.0001) scores. Crustaceans scored higher than finfish on NRF6.2 (*p* < 0.0001) and NRF9.2 (*p* = 0.005).

### 3.2 Nutrient density of mollusks by subgroup

Table 1 also compares mollusk subgroups (bivalve, cephalopod, gastropod). Bivalves were highest in iron, significantly higher than all other groups (all *p* < 0.0001) besides gastropods (*p* = 0.4211). Bivalves were significantly higher in zinc compared to all other groups (all *p* < 0.0001) except for gastropods (*p* = 0.1130). Gastropods contained the most calcium and magnesium (all *p* < 0.0001). Cephalopods were high in vitamin A and vitamin B12.

The high nutrient density of mollusks was reflected by NRF nutrient density scores. Bivalves had the highest NRF6.2 and NRF9.2 nutrient density scores. Bivalves scored higher than any other group on NRF6.2 (all *p* < 0.0001). Cephalopods scored higher on the NRF6.2 compared to gastropods (*p* < 0.05). Gastropods had the lowest nutrient density scores.

### 3.3 Nutrient density of finfish

Unlike crustaceans and mollusks, finfish contained significant amounts of vitamin D (see Table 2). Small pelagic fish were significantly higher in vitamin D than any other group (all *p* < 0.0001) Small pelagic fish and salmonids contained more omega-3 fatty acids than any other group (all *p* < 0.0001). Small pelagic fish were also high in selenium (all *p* < 0.0001). Among finfish, all groups were similar in vitamin A content besides cod, which was significantly lower in vitamin A compared to salmonids (*p* = 0.0072) and demersal fish (*p* = 0.0169). There were no significant differences in magnesium between finfish groups (all *p* > 0.05).

Table 2 also shows mean NRF6.2 and NRF9.2 scores. Small pelagic fish scored similarly to crustaceans (*p* = 0.4791) and cephalopods (*p* = 0.1927) on the NRF6.2. Small pelagic fish had the highest NRF9.2 scores, well above any other group (all *p* < 0.0001).

**Table 2:** Nutrient content per 100 g of Finfish by taxonomic group.

Nutrient	Cichlid (*N* = 24)		Cod (*N* = 48)		Demersal (*N* = 96)		Small pelagic (*N* = 12)		Salmonid (*N* = 69)		*P*-value
	Mean	SD	Mean	SD	Mean	SD	Mean	SD	Mean	SD	
Protein (g)	20.9^cd^	2.25	21.6^cd^	2.58	20.7^cd^	2.91	23.8^bcd^	3.51	22.5^c^	2.03	<0.0001
Iron (mg)	1.02^cd^	1.01	0.20^d^	0.07	0.71^cd^	0.62	1.24^cd^	0.19	0.45^d^	0.19	<0.0001
Zinc (mg)	1.85^b^	2.83	0.45^b^	0.07	1.12^b^	1.80	0.52^b^	0.09	0.57^b^	0.23	<0.0001
Calcium (mg)	40.8^d^	45.5	16.2^d^	7.41	27.7^d^	13.4	38.4^cd^	12.0	17.7^d^	7.53	<0.0001
Potasssium (mg)	321^bc^	43.6	394^a^	67.5	337^b^	65.2	433^a^	67.1	402^a^	58.3	<0.0001
Magnesium (mg)	30.1^cd^	4.52	29.0^d^	4.09	27.6^d^	4.95	35.2^cd^	5.23	28.3^d^	3.13	<0.0001
Selenium (mcg)	29.3^c^	7.07	30.8^c^	5.53	27.9^c^	14.0	66.2^a^	8.60	23.6^c^	4.99	<0.0001
Folate (mg)	12.5^bc^	1.13	9.65^c^	2.13	10.2^c^	2.21	2.13^c^	0.19	8.34^c^	3.55	<0.0001
Vit. B12 (mcg)	1.31^d^	0.21	1.47^d^	0.48	4.10^cd^	3.88	7.81^bc^	0.74	4.44^cd^	0.87	<0.0001
Vit. A (mcg)	2.63^de^	3.61	1.89^e^	0.57	12.4^cd^	8.20	4.80^cde^	0.59	13.8^cd^	4.34	<0.0001
Vitamin D (mcg)	21.0^b^	1.07	1.44^d^	0.77	1.50^d^	1.31	30.1^a^	3.75	9.23^c^	3.21	<0.0001
Omega-3 (g)	0.16^c^	0.10	0.26^c^	0.10	0.37^b^	0.30	1.56^a^	0.88	1.89^a^	1.02	<0.0001
NRF6.2	75.5^cd^	37.0	69.3^d^	21.1	89.6^c^	39.9	103^bcd^	2.85	90.5^c^	15.3	<0.0001
NRF9.2	219^bc^	17.2	153^de^	14.6	120^f^	39.9	289^a^	9.39	152^e^	28.6	<0.0001

Means within rows that do not share a superscript letter are significantly different at the *p* < 0.05 level based on Tukey’s HSD post hoc tests.

### 3.4 Nutrient density of wild versus farmed fish

Table 3 shows that wild fish were significantly more rich in iron, zinc, vitamin B12, potassium, magnesium, and selenium than farmed finfish (all *p* < 0.05). Farmed fish were significantly higher in vitamin A (*p* = 0.022) and total fat (*p* < 0.0001). Farmed and wild finfish contained similar levels of calcium, folate, vitamin D, and omega 3. However, wild fish had a significantly higher ratio of omega-3 to total fat (*p* < 0.0001). Overall, wild fish scored higher than farmed fish on both the NRF6.2 (*p* < 0.0001) and NRF 9.2 (*p* < 0.0001) (Table 3).

**Table 3:** Nutrient content of wild and farmed finfish.

Nutrient	Farmed (*N* = 132)		Wild (*N* = 117)		*P*-value
	Mean	SD	Mean	SD	
Protein (g)	21.30	2.79	21.80	2.67	0.146
Iron (mg)	0.52^b^	0.31	0.68^a^	0.77	0.036*
Zinc (mg)	0.60^b^	0.30	1.19^a^	209	0.002*
Calcium (mg)	22.31	19.10	26.55	17.12	0.072
Potassium (mg)	350.48^b^	66.59	389.60^a^	70.49	<0.0001*
Magnesium (mg)	26.97^b^	3.73	30.52^a^	4.77	<0.0001*
Selenium (mcg)	24.51^b^	9.83	34.62^a^	14.27	<0.0001*
Folate (mcg)	9.28	3.15	9.55	3.31	0.510
Vitamin B12 (mcg)	2.94^b^	1.48	4.34^a^	3.85	0.0001*
Vitamin A (mcg)	10.47^a^	5.36	8.38^b^	9.37	0.022*
Vitamin D (mcg)	6.50	6.61	7.33	9.82	0.434
Omega-3 (g)	0.88	0.99	0.72	0.87	0.1842
Total fat (g)	6.90^a^	4.96	3.24^b^	3.57	<0.0001*
Omega-3: Total fat	0.12^b^	0.10	0.25^a^	0.09	<0.0001*
NRF6.2	75.90^b^	30.64	95.69^a^	28.47	<0.0001*
NRF9.2	64.91^b^	36.94	85.77^a^	33.94	<0.0001*

Superscripts and * indicate significant differences between mean nutrient values at the *p* < 0.05 level in farmed versus wild finfish based on one-way ANOVA tests.

### 3.5 Nutrient density by broad taxonomic group

**Figure 1:**
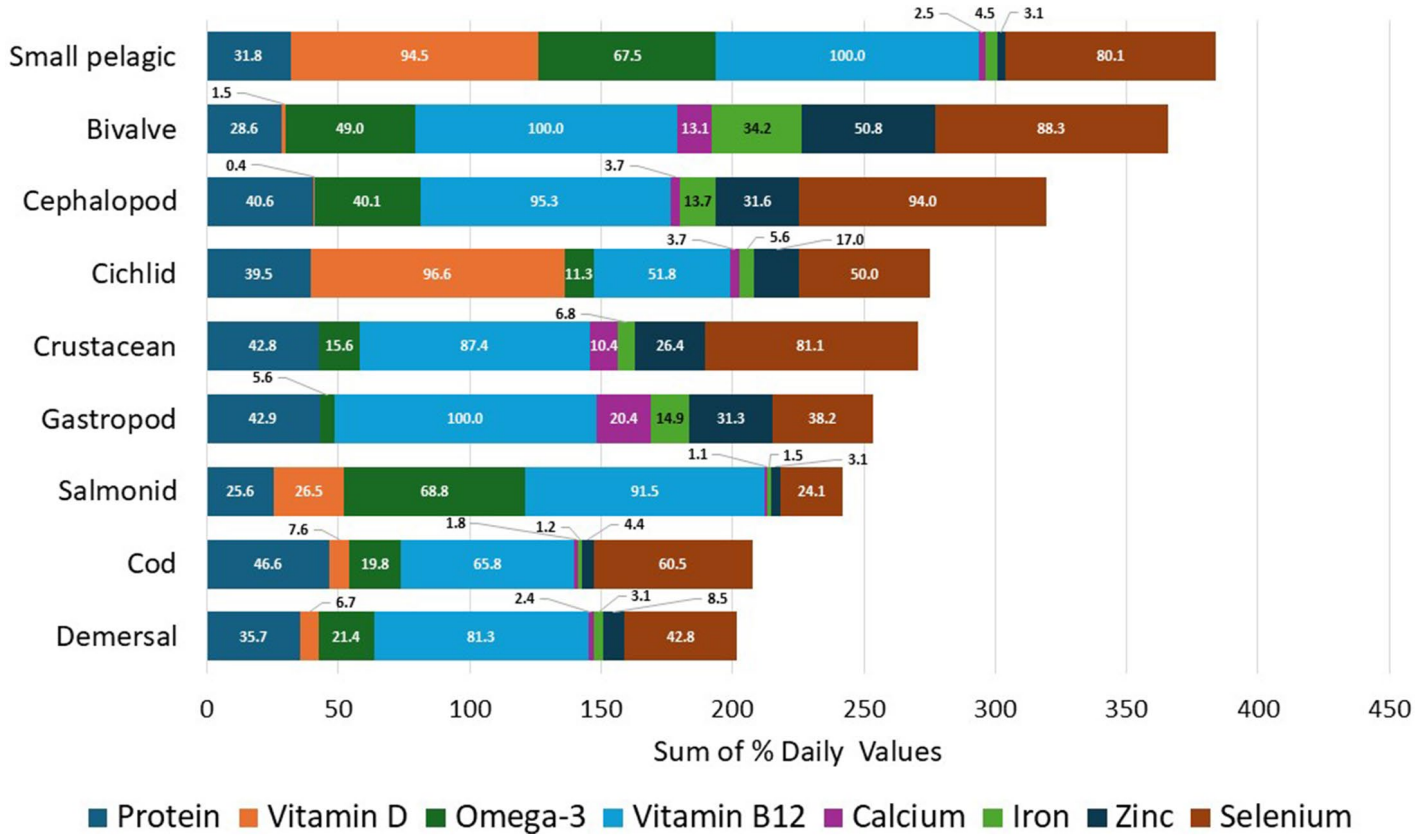
Mean %DV protein and micronutrients provided by broad taxonomic group.

Figure 1 shows that all groups provided >20% DV per 100 g of selenium, vitamin B12, and protein. Bivalves, gastropods, cephalopods, and crustaceans provided >20% DV for zinc. Only gastropods had >20% DV for calcium. Small pelagic fish, cichlids, and salmonids provided >20% DV of vitamin D. Bivalves, cephalopods, demersal fish, small pelagic fish, and salmonids provided >20% DV of omega-3 fatty acids. Bivalves provided >20% DV for iron.

### 3.6 Nutrient density of seafood assessed using NRF6.2 and NRF9.2 scores

Table 4 shows rank-ordered NRF6.2 and NRF9.2 scores by taxonomic group and by species. Oysters scored the highest on the NRF6.2, significantly higher than any other species (*p* < 0.0001) besides clams (*p* = 0.877). Oysters, clams and mussels had high NRF9.2 scores, but mackerel scored significantly higher than any other species (all *p* < 0.0001) besides clams (*p* = 1.000). Small pelagic fish had the highest NRF9.2 scores. Bivalves, including clams, oysters, and mussels, fall shortly behind mackerel. Tilapia had very high NRF9.2 scores, significantly above all species (*p* < 0.05) and equal to mackerel, clams, oysters, mussels, octopus and squid.

**Table 4:** Ranking by NRF6.2 and NRF9.2.

Group Ranking	Highest NRF6.2				Highest NRF9.2			
	Name	Mean	*N*	SEM	Name	Mean	*N*	SEM
1	Bivalve	192.46	66	6.16	Small pelagic	288.80	12	2.71
2	Cephalopod	131.74	36	4.03	Bivalve	229.38	66	5.61
3	Crustacean	123.76	152	2.74	Cichlid	219.19	24	3.51
4	Small pelagic	102.54	12	0.82	Cephalopod	207.45	132	2.53
5	Salmonid	90.53	69	1.85	Crustacean	167.19	152	2.21
6	Demersal	89.55	96	4.07	Cod	153.33	48	2.10
7	Gastropod	84.80	12	4.66	Salmonid	151.69	69	3.45
8	Cichlid	75.52	24	7.55	Gastropod	149.97	12	8.02
9	Cod	69.33	48	3.05	Demersal	120.27	96	4.07

Species ranking	Highest NRF6.2				Highest NRF9.2			
	Name	Mean	*N*	SEM	Name	Mean	*N*	SEM
1	Oysters	228.72	27	8.33	Mackerel	288.80	12	2.71
2	Clams	201.69	6	3.24	Clams	277.57	6	9.31
3	Mussels	169.74	27	7.50	Oysters	242.66	27	4.32
4	Crab	160.18	40	1.44	Mussels	227.69	27	7.54
5	Octopus	157.04	12	4.38	Tilapia	219.19	24	3.51
6	Crayfish	137.27	12	3.25	Squid	207.62	24	3.78
7	Scallops	126.77	6	0.78	Octopus	207.10	12	1.29
8	Squid	119.09	24	3.41	Crab	182.93	40	3.09
9	Pike	114.62	41	0.50	Crayfish	176.07	12	4.53
10	Shrimp/Prawn	114.30	76	3.29	Lobster	172.76	24	3.02
11	Abalone	111.50	3	0.34	Conch	164.66	9	2.98
12	Bass	105.64	40	1.12	Pike	164.07	41	9.22
13	Mackerel	103.53	12	0.82	Salmon	162.13	41	4.25
14	Trout	100.09	28	2.26	Shrimp/Prawn	155.75	76	3.46
15	Lobster	86.26	24	4.40	Cod	153.33	48	2.10
16	Salmon	84.00	41	2.19	Bass	140.02	40	1.98
17	Catfish	82.74	64	5.79	Sole	139.23	8	2.07
18	Sole	82.34	8	4.94	Trout	136.40	28	4.46
19	Conch	75.89	9	0.50	Scallops	129.01	6	2.87
20	Tilapia	75.52	24	7.55	Abalone	105.88	3	3.62
21	Cod	69.33	48	3.05	Catfish	105.99	64	4.87

**Figure 2:**
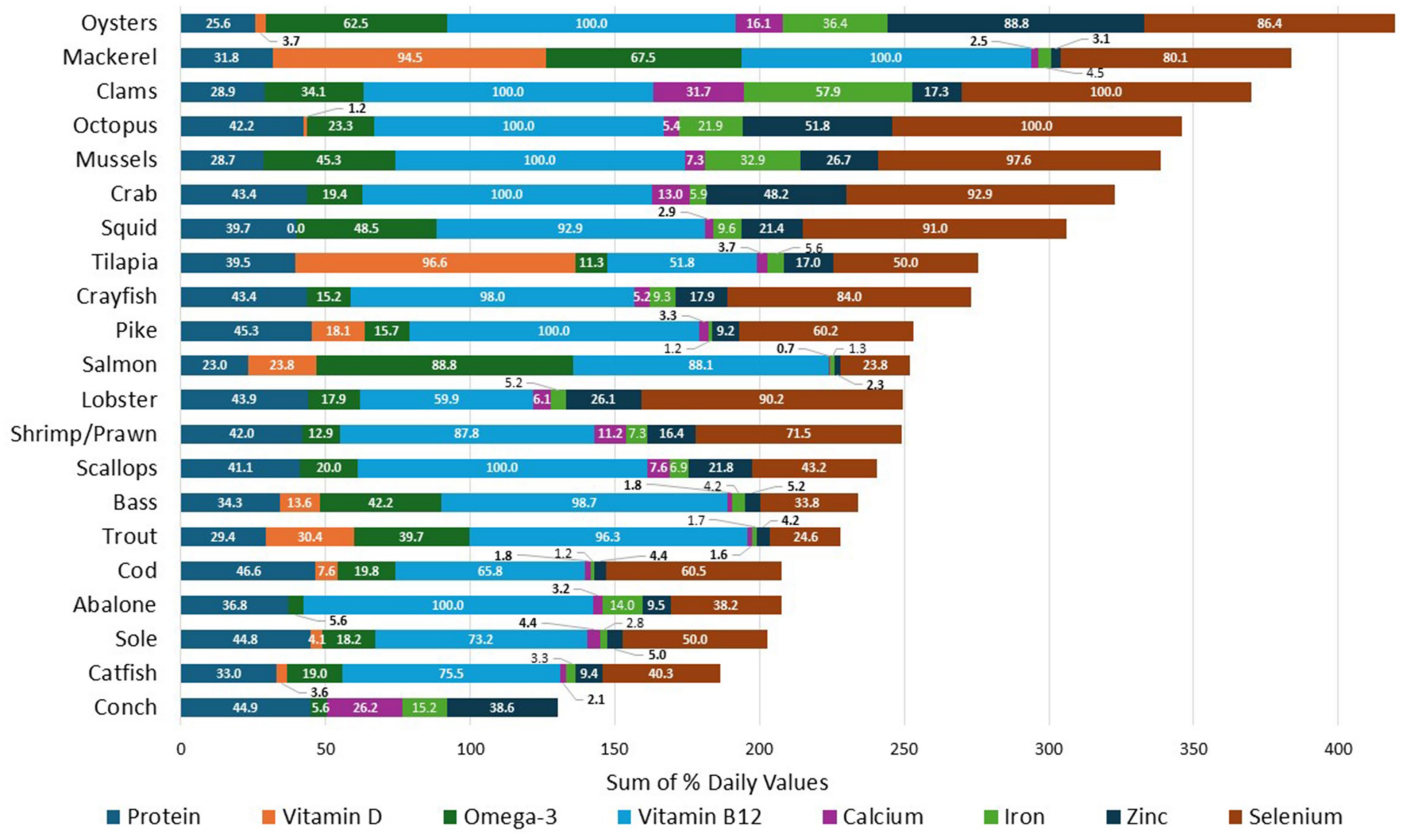
Mean % DV protein and micronutrients per 100g of each species group.

Figure 2 shows %DVs per 100 g by species group. Additional data for specific nutrients are provided in Supplementary Table 2. Conch, mussels, octopus, and oysters were high in both iron and zinc. Oysters were significantly higher in zinc than any other species (all *p* < 0.0001). Clams, mussels, conch, oysters, and octopus contained more iron than most other species (all *p* < 0.05) Conch provided more magnesium than any other species (all *p* < 0.0001). All species were high in selenium and vitamin B12, with clams having the highest content (all *p* < 0.0001). Octopus, squid, mussels and mackerel contained significantly more selenium than all species (all *p* < 0.05) besides clams. Octopus and oysters were significantly higher in vitamin B12 than all species (all *p* < 0.0001) other than lobster (Supplementary Table 2).

Only finfish such as mackerel, salmon, tilapia, and trout were excellent sources of vitamin D. Mackerel and tilapia were higher in vitamin D than any other species (all *p* < 0.0001). All bivalve and cephalopod species were excellent sources of omega-3 s fatty adicds as were finfish. Salmon and mackerel had the highest content of omega-3 fatty acids compared to other species (*p* < 0.05) (Supplementary Table 2).

### 3.7 The cost of 100 g protein and NRF6.2- and NRF9.2-affordability indices

Table 5 shows that conch, tilapia, mackerel, squid, and mussels were the lowest cost protein sources. The next lower cost sources of protein were bass, octopus, and cod. Pike, sole, scallops, and catfish were more expensive. Crab and shrimp/prawn were more expensive still. The least affordable sources of protein were lobster and oysters. Also shown in Table 5 are the calculated mean NRF6.2 and NRF 9.2 affordability indices by species group. Mussels have the highest NRF6.2 affordability index. Tilapia, squid and mackerel all score similarly high on the NRF6.2 affordability index following mussels. Tilapia, mackerel and mussels have the highest NRF9.2 affordability indices.

**Table 5:** Mean price per kg, yield, cost per 100 g protein and affordability indices for aquatic foods by broad taxonomic group and species.

Broad taxonomic group	Species group	Price $/kg*	Yield**	Cost $/100 g protein	NRF6.2 affordability index	NRF9.2 affordability index
Crustacean	Crab	13.33	0.25	28.36	3.00	3.43
	Lobster	34.44	0.28	59.72	0.70	1.41
	Shrimp/Prawn	27.96	0.46	28.53	1.88	2.57
	Crayfish	N/A	0.12	N/A	N/A	N/A
Demersal	Bass	6.74	0.39	7.32	6.13	8.10
	Catfish	6.50	0.19	17.36	2.42	3.10
	Pike	9.81	0.38	11.84	4.46	6.35
Cod	Cod	7.53	0.38	9.18	3.50	7.72
Small pelagic	Mackerel	4.44	0.54	3.45	12.54	35.18
Salmonid	Trout	7.19	0.66	4.98	6.38	12.31
	Salmon	8.56	0.65	5.75	9.17	12.48
Cichlid	Sole	19.71	0.60	14.60	2.51	4.23
	Tilapia	5.10	1.00	2.45	14.79	42.90
Gastropod	Abalone	N/A	0.42	N/A	N/A	N/A
	Conch	7.09	0.68	2.74	7.24	15.74
Bivalve	Clams	14.72	0.20	57.48	2.75	3.78
	Mussels	3.51	0.51	4.53	24.70	33.13
	Oysters	17.95	0.11	168.20	1.40	1.49
	Scallops	5.30	0.13	16.49	3.11	3.16
Cephalopod	Octopus	13.11	0.79	7.41	9.46	12.47
	Squid	7.38	0.78	3.56	12.58	21.99

*Prices from GlobeFish; **yield from USDA Handbook 102.

**Figure 3:**
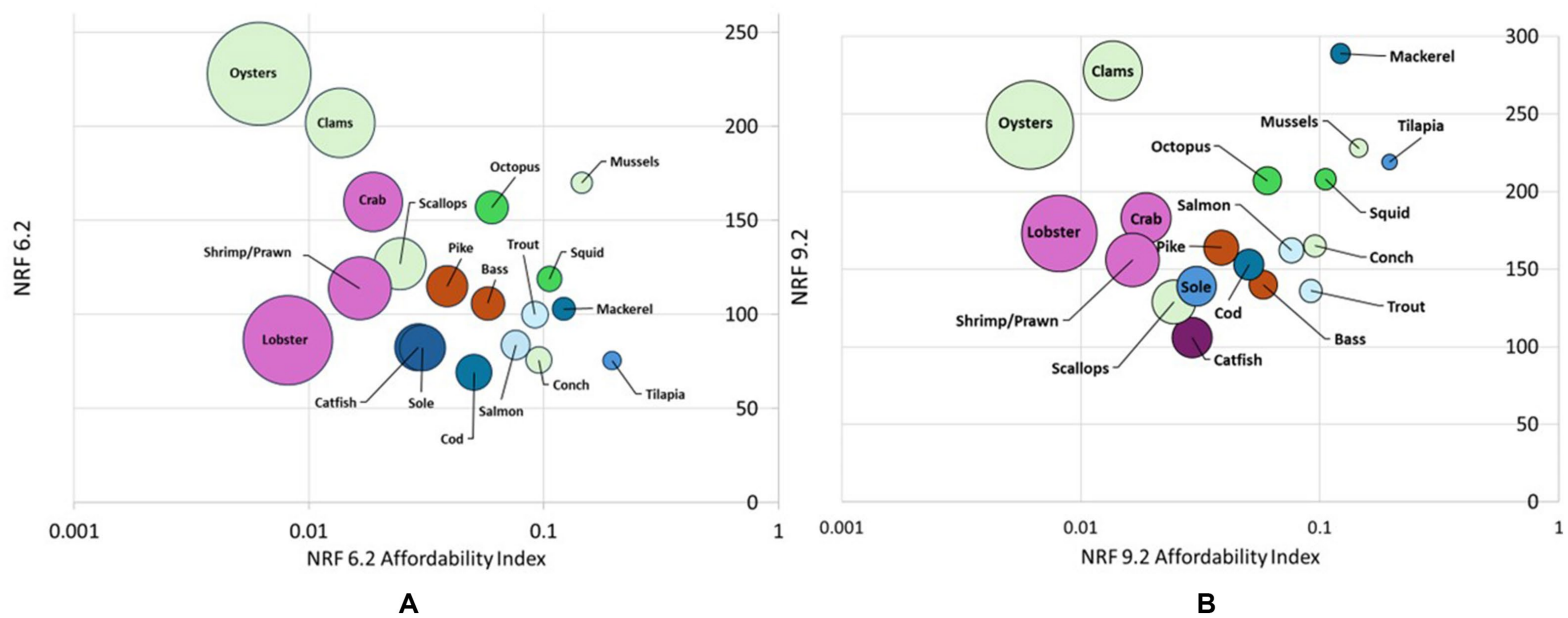
**(A)** Bubble plot of NRF6.2 versus affordability by species group and **(B)** Bubble plot of NRF9.2b versus affordability by species group. Note. The x-axis uses a logarithmic scale. Bubble sizes are proportional to price per kg (after yield correction).

Figure 3 demonstrates the tradeoffs between nutrient density and price of aquatic foods by plotting NRF nutrient density scores against the Affordability Index shown here on a logarithmic scale. The bubble size is proportional to the price per edible portion.

Figure 3A plots affordability against the mean NRF6.2 score by species group, illustrating an inverse relationship between priority micronutrient density and affordability. Although bivalves scored highest on the NRF6.2, oysters, clams, and scallops were among the least affordable aquatic foods. Mussels are an exception, coming at a lower mean price and higher edible portion. When taking affordability into account, mussels, tilapia, mackerel and squid provide the most NRF6.2 nutrients per penny (Supplementary Table 3).

Figure 3B plots affordability against the mean NRF9.2 by species group. Figure 3A suggests an inverse relationship between affordability and nutrient density, conversely, Figure 3B reveals that some of the most affordable aquatic foods are also the densest in NRF9.2 nutrients. Mussels, tilapia, mackerel, and squid scored similarly well on the NRF9.2 as oysters and clams but are much more affordable. As a result, mussels, tilapia, mackerel and squid provide the most NRF9.2 nutrients per penny in addition to providing the most NRF6.2 nutrients per penny (Supplementary Table 3).

**Figure 4:**
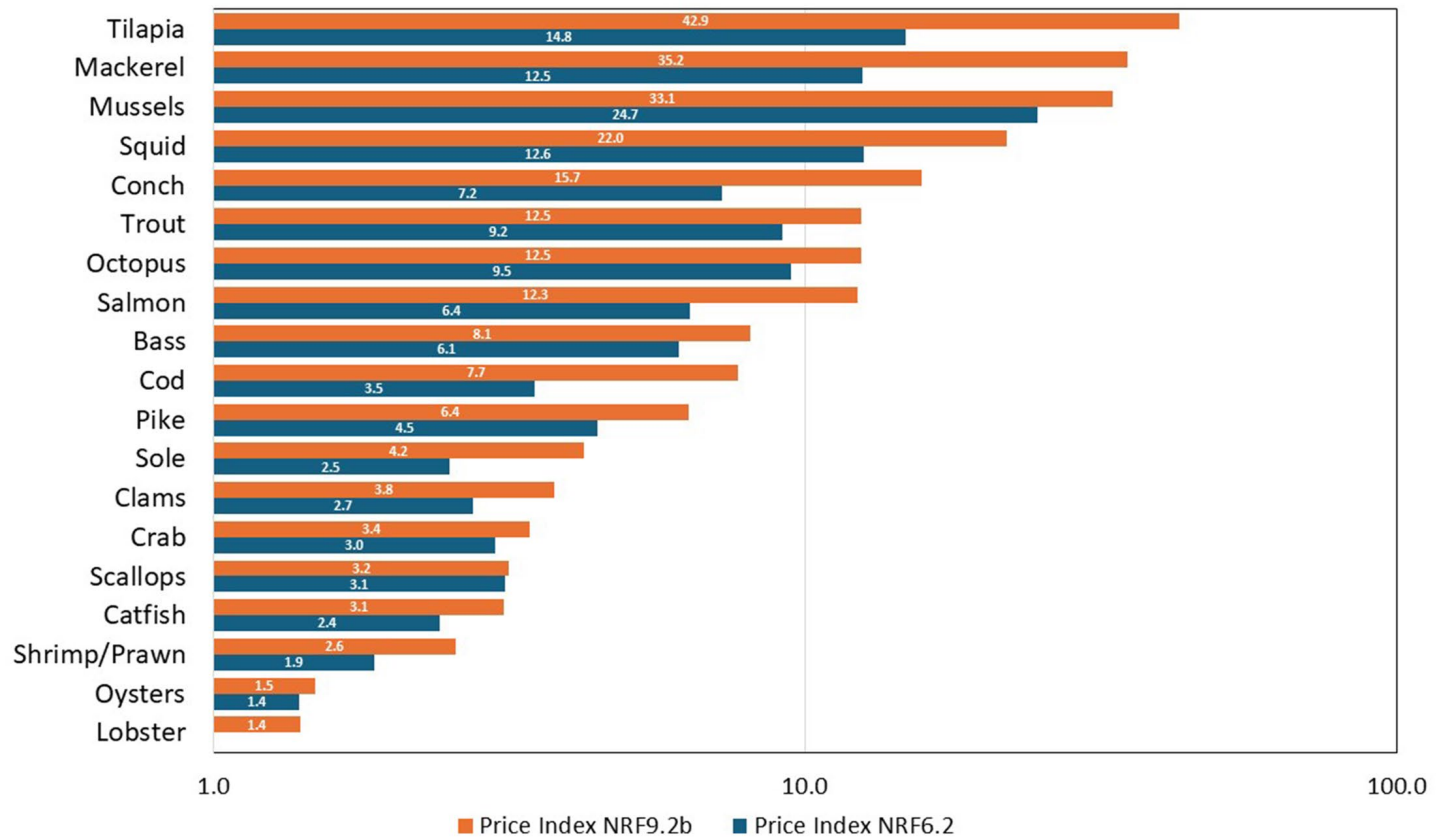
NRF Affordability indices by species group. Note. The x-axis is logarithmic.

Figure 4 is a bar plot displaying the NRF affordability indices by species group, which helps to visually summarize the trends described above.

## 4 Discussion

In this analysis, mollusks had the highest content of priority micronutrients. Bivalves, including oysters, clams, and mussels had the highest NRF6.2 scores. This is consistent with the observations of Beal et al. which identified bivalves, in addition to crustaceans, canned fish with bones, and dried small fish as top sources of priority micronutrients (2). Beal et al. found that bivalves were the only food with at least moderate density of all six priority micronutrients (2). Of note, the uFISH database includes raw, cooked, and smoked fresh aquatic foods, but not dried or canned small fish with bones, which are more nutrient dense (2).

Mollusks also had higher average NRF9.2 scores compared to finfish and crustaceans. However, dividing finfish, mollusks, and crustaceans into smaller groups revealed that small pelagic fish scored highest, surpassing bivalves. These findings are consistent with Hallström et al., which also identified small pelagic fish and bivalves as the most nutrient-rich, using similar methods on the Swedish food database (19). Bianchi et al. (20) similarly used the NRF algorithm to assess fish and shellfish, using multiple databases, including uFISH, finding that oysters and small pelagic fish ranked highest based on nutrient density. Small pelagic fish species and wild caught pink salmon ranked the highest based on combined climate and nutritional impact (20). Golden et al. compared aquatic and terrestrial source foods and found that aquatic foods were higher in priority micronutrients with small pelagic fish, bivalves, and salmonids being the most nutrient rich (21).

Although mollusks and crustaceans were highly nutrient-dense, they provided the least nutrients per penny due to their high cost per edible portion. Two exceptions were mussels and squid which were affordable sources of micronutrients. The present analyses clearly demonstrate that seafood can be an affordable source of protein, omega-3, and important micronutrients. Mackerel, tilapia, mussels, and squid provided the highest NRF6.2 and NRF9.2 nutrients per penny as they were both nutrient dense and affordable. Mackerel, tilapia, squid, and mussels also provided the most protein per penny.

Wild finfish had higher mean NRF6.2 and NRF9.2 scores compared to farmed fish. This may be because the wild finfish category included nutrient-rich small pelagic fish. Small pelagic fish are forage fish that are a major component of fishmeal and fish oil used to feed predatory finfish grown in aquaculture. Willer et al. compared the nutrient composition of Atlantic salmon to the small pelagic fish that are used to feed them and found that vitamin D, zinc, and selenium were retained while vitamin B12, vitamin A, Omega-3, iron, iodine and calcium were higher in the small pelagic fish used for fish feed than in farmed salmon (22). This represents a net loss of micronutrients available for human consumption. Farmed species are consumed at higher rates in higher income countries while lower income countries have historically relied more on smaller, more nutrient-dense and affordable fish species.

The FAO roadmap for the “Blue Transformation” is intended to guide the expansion of global sustainable aquaculture production by at least 35 percent by 2030, in alignment with the Sustainable Development Goal (SDG) to end hunger, achieve food security and improved nutrition, and promote sustainable agriculture (23). The growth of aquaculture requires effective management and oversight to ensure that resources are distributed equitably and that guidelines are followed to ensure social, environmental, and economic sustainability. The FAO’s stated mission is to expand aquaculture while reducing fish loss and waste, increasing seafood consumption across the global south, supporting local economies, and improving gender equity across the aquatic food value-chain (24). Priority actions include supporting small and medium scale fish farmers through information, technology, and policy. The most recent update on the state of world fisheries and aquaculture came in October 2024. Progress has been made in many areas while other areas have drifted from the target, including the biologically sustainable management of fish stocks.

Aquaculture remains dominated by a small number of countries (25). In many low- and middle-income countries (LMIC), small pelagic fish caught by small-scale fisheries that support local economies while providing an affordable source of micronutrients and protein that can be made accessible year round through canning, drying, salting, and smoking (26). Aquatic foods are rich in micronutrients that are difficult to obtain from plant-based sources. Small fish have the potential to fill the micronutrient gap for calcium, selenium, and zinc for millions of women in LMIC in Africa and Asia (27).

The study is one of the first to systematically assess nutrient density and affordability of aquatic foods using the FAO/INFOODS uFISH database, filling an important gap in nutrition and sustainable diets research. The uFISH database provides data for a broader range of seafood species than previously available, allowing comparison between several categories of finfish, shellfish, cephalopods, and crustaceans. The use of Nutrient Rich Food (NRF) indices, specifically the tailored NRF6.2 and NRF9.2 scores for aquatic foods, is innovative and appropriate for highlighting species-level differences in micronutrient content. The methodology integrates composition data with food price data, allowing a robust analysis of both nutritional value and affordability—critical for informing policy and public health interventions in LMICs.

The statistical analysis is comprehensive, employing one-way ANOVA, post-hoc tests, and careful reporting of significance, which strengthens the reliability of findings.

There are several limitations to the ability to capture affordable nutrient density of fish and shellfish using the uFISH and Globefish databases. Some data gaps exist in the uFISH database, necessitating imputation from other sources (11, 13). This may limit the representativeness and accuracy for certain species. The study does not include dried or canned small fish with bones, which are highly nutrient-dense and could alter affordability and nutrient density rankings. The expansion of the FAO database would allow for more comprehensive analysis and comparison of aquatic species. Aquatic plant species would be a valuable addition to the uFISH database. Currently, algae and other phytoplankton, in addition to corn, soy, and insects, are being utilized in fish feed as alternatives to wild fish, influencing the nutritional properties of the final product. Aquatic plants are often overlooked as valuable sources of micronutrients, EPA, and DHA. Expanding their use for direct human consumption could offer a sustainable way to address micronutrient gaps. The price data is derived from GlobeFish European markets in October 2024. Affordability indices may not accurately reflect global or local market conditions, especially in LMICs, and may fluctuate seasonally. Accessibility is discussed separately from affordability; trade patterns and local supply are not quantitatively assessed, which limits practical recommendations for specific populations.

An analysis of trade patterns by Nash et al. found that international trade diverts nutrients caught in marine fisheries from nutrient-insecure LMIC toward wealthy, nutrient-secure countries (28). Ideally, distribution processes should match food quality with dietary deficiencies and food security and micronutrient deficiencies should be considered when developing trade agreements. Future directions for this research include tailoring nutrient affordability indices to reflect the accessibility and affordability of aquatic foods to vulnerable populations in LMIC. Nutrient profiling models may also be further tailored to reflect the specific needs of vulnerable groups in different countries or regions to direct food system solutions that aim to increase the production and accessibility of fish and shellfish.

## 5 Conclusion

Aquatic foods have the potential to close gaps in dietary micronutrients globally. Efforts should focus on targeted food systems solutions that improve and preserve the accessibility and affordability of nutrient rich aquatic foods to vulnerable populations. The nutrient affordability index is a valuable tool to weigh the trade-offs between nutrient richness and cost, which is essential for guiding policy and public health interventions in LMICs. This analysis highlights mackerel, tilapia, squid, and mussels as affordable sources of protein and key micronutrients, offering actionable targets for nutrition policies and interventions aimed at reducing protein-calorie malnutrition and micronutrient deficiencies worldwide.

## Data Availability

Publicly available datasets were analyzed in this study. This data can be found at: https://www.fao.org/infoods/infoods/tables-and-databases/faoinfoods-databases/en/.

## References

[1] Passarelli S Free CM Shepon A Beal T Batis C Golden CD. Global estimation of dietary micronutrient inadequacies: a modelling analysis. Lancet Glob Health. (2024) 12:e1590–9. doi: 10.1016/S2214-109X(24)00276-6, PMID: 39218000 PMC11426101

[2] Beal T Ortenzi F. Priority micronutrient density in foods. Front Nutr. (2022) 9:806566. doi: 10.3389/fnut.2022.806566, PMID: 35321287 PMC8936507

[3] Weffort VRS Lamounier JA. Hidden hunger – a narrative review. J Pediatr. (2023) 100:S10–7. doi: 10.1016/j.jped.2023.08.009 PMC10960185 37918810

[4] Jiang W Zhao Y Wu X Du Y Zhou W. Health inequalities of global protein-energy malnutrition from 1990 to 2019 and forecast prevalence for 2044: data from the global burden of disease study 2019. Public Health. (2023) 225:102–9. doi: 10.1016/j.puhe.2023.10.003, PMID: 37924634

[5] Byrd KA Shieh J Mork S Pincus L O’Meara L Atkins M. Fish and fish-based products for nutrition and health in the first 1000 days: a systematic review of the evidence from low and middle-income countries. Adv Nutr. (2022) 13:2458–87. doi: 10.1093/advances/nmac102 36166842 PMC9776644

[6] Viana DF Zamborain-Mason J Gaines SD Schmidhuber J Golden CD. Nutrient supply from marine small-scale fisheries. Sci Rep. (2023) 13:11357. doi: 10.1038/s41598-023-37338-z, PMID: 37443165 PMC10344920

[7] FAO. Apparent consumption of aquatic foods. (2024).

[8] INFOODS: FAO/INFOODS Databases. (2016). Available online at: https://www.fao.org/infoods/infoods/tables-and-databases/faoinfoods-databases/en/ (Accessed February 23, 2025).

[9] FAO. GLOBEFISH | European Price report October 2024. FAOorg (2024). Available online at: https://openknowledge.fao.org/items/4630a189-e96b-402d-a5b0-77b979dd63b5 (Accessed February 18, 2025).

[10] Herforth A Bai Y Mahrt K Ebel A. Cost and affordability of healthy diets across and within countries. Rome: FAO (2020).

[11] The National Food Institute. Technical University of Denmark. Frida - The Danish Food Composition Database. (2024). Available online at: https://frida.fooddata.dk/data? (Accessed February 18, 2025).

[12] MEXT. Standard tables of food composition in Japan. (2015). Available online at: https://www.mext.go.jp/en/policy/science_technology/policy/title01/detail01/1374030.htm (Accessed February 18, 2025).

[13] USDA, ARS. USDA food and nutrient database for dietary studies 2021-2023. Food Surv Res Group Home Page. (2024). Available online at: http://www.ars.usda.gov/nea/bhnrc/fsrg (Accessed February 18, 2025).

[14] Beal T Massiot E Arsenault JE Smith MR Hijmans RJ. Global trends in dietary micronutrient supplies and estimated prevalence of inadequate intakes. PLoS One. (2017) 12:e0175554. doi: 10.1371/journal.pone.0175554, PMID: 28399168 PMC5388500

[15] Durazzo A Di Lena G Gabrielli P Santini A Lombardi-Boccia G Lucarini M. Nutrients and bioactive compounds in seafood: quantitative literature research analysis. Fishes. (2022) 7:132. doi: 10.3390/fishes7030132

[16] Program HF. Daily value on the nutrition and supplement facts labels. FDA (2024). Available online at: https://www.fda.gov/food/nutrition-facts-label/daily-value-nutrition-and-supplement-facts-labels (Accessed April 19, 2025).

[17] Federal Reserve. Foreign Exchange Rates - H.10. Federalreservegov. Available online at: https://www.federalreserve.gov/releases/h10/hist/dat00_eu.htm (Accessed February 23, 2025).

[18] USDA Agricultural Resarch Service. USDA agriculture handbook no. 102. USDA ARS. (1975). Available online at: https://www.fao.org/uploads/media/USDA_handbook_no8_02.pdf (Accessed February 23, 2025).

[19] Hallström E Bergman K Mifflin K Parker R Tyedmers P Troell M. Combined climate and nutritional performance of seafoods. J Clean Prod. (2019) 230:402–11. doi: 10.1016/j.jclepro.2019.04.229

[20] Bianchi M Hallström E Parker RWR Mifflin K Tyedmers P Ziegler F. Assessing seafood nutritional diversity together with climate impacts informs more comprehensive dietary advice. Commun Earth Environ. (2022) 3:1–12. doi: 10.1038/s43247-022-00516-4

[21] Golden CD Koehn JZ Shepon A Passarelli S Free CM Viana DF. Aquatic foods to nourish nations. Nature. (2021) 598:315–20. doi: 10.1038/s41586-021-03917-1, PMID: 34526720 PMC10584661

[22] Willer DF Newton R Malcorps W Kok B Little D Lofstedt A. Wild fish consumption can balance nutrient retention in farmed fish. Nat Food. (2024) 5:221–9. doi: 10.1038/s43016-024-00932-z, PMID: 38509235 PMC10963266

[23] SDG Goal 2: Zero Hunger. UNICEF DATA. Available online at: https://data.unicef.org/sdgs/goal-2-zero-hunger/ (Accessed December 30, 2024).

[24] Blue transformation - roadmap 2022–2030. FAO. (2022). doi: 10.4060/cc0459en

[25] FISH4ACP. FAO>org. (2025). Available online at: https://www.fao.org/in-action/fish-4-acp/where-we-work/en/ (Accessed December 29, 2024).

[26] FAO. The State of World Fisheries and Aquaculture. FAO. (2024). 191–195. doi: 10.4060/cd0683en

[27] University D. WorldFish, Illuminating hidden harvests. FAO; Duke University; WorldFish. (2023). https://openknowledge.fao.org/handle/20.500.14283/cc4576en (Accessed January 12, 2025).

[28] Trade and foreign fishing mediate global marine nutrient supply | PNAS. Available online at: https://www.pnas.org/doi/abs/10.1073/pnas.2120817119 (Accessed May 26, 2025) 10.1073/pnas.2120817119 PMC9295801 35605118

