# Supplementary material for: Nutrient density and affordability of aquatic foods in the FAO uFISH database assessed using Nutrient Rich Food (NRF) indices
Source: Front Nutr. 2025 Nov 3;12:1675142. doi: 10.3389/fnut.2025.1675142 (PMC12621452; doi:10.3389/fnut.2025.1675142)
Supplement: Supplementary file 1 [file Table_1.docx]

**Supplementary Tables and Figures: Nutrient Density and Affordability of Aquatic Foods in the FAO uFISH Database Assessed by Nutrient Rich Food (NRF) Indices; Emma Johnsson, Cristen Harris, Adam Drewnowski**

##### **Supplementary Table 1.** Classification of Aquatic Foods

| Phylum, Subphylum, or Biological Distinction | Zone | Family or Order | Species grouping | Species in uFISH (Common names) |
| --- | --- | --- | --- | --- |
| **Finfish** | **Demersal** |  | **Bass** | European sea bass |
|  |  |  | **Catfish** | Torpedo-shaped catfish, Philippine catfish, North African catfish, Bighead catfish, Channel catfish, Striped catfish |
|  |  |  | **Pike** | Northen Pike |
|  |  | **Cods** | **Cod** | Atlantic cod, pacific cod |
|  | **Pelagic** |  | **Mackerel** | Atlantic horse mackerel |
|  | Pelagic (migratory) | **Salmonid** | **Salmon** | Atlantic salmon |
|  |  |  | **Trout** | Rainbow trout |
|  | Variable | **Cichlid** | **Sole** | Common sole |
|  |  |  | **Tilapia** | Nile tilapia |
| **Crustacean** | Class | | Species grouping | Species in uFISH (Common names) |
|  | Malacostraca | | **Crab** | Rock crab, Dungeness crab, Edible crab, Tanner crab, Swimming crab, Blue crab, Blue swimming crab, Indo-Pacific swamp crab, King crab, Red king crab |
|  |  |  | **Crayfish** | Oceanian crayfish |
|  |  |  | **Lobster** | Lobsterettes, American lobster, European lobster, Norway lobster, Spiny lobster, Red rock lobster |
|  |  |  | **Shrimp/ Prawn** | Common shrimp, Pandalus shrimp, Penaeid shrimp, Endeavour shrimp, Eastern school shrimp, Speckled shrimp, Whiteleg shrimp, Sergestid shrimp, Palaemonid shrimp; River prawn, Monsoon River prawn, Giant River prawn, Northen prawn, Brown tiger prawn, Western king prawn, Banana prawn, Giant tiger prawn, Green tiger prawn |
| **Mollusk** | **Bivalve** | | **Clams** | Venus clams |
|  |  |  | **Mussels** | Mytilus mussel, Korean mussel, Blue mussel, Mediterranean mussel, Perna mussel, New zealand mussel, Green mussel |
|  |  |  | **Oysters** | Cupped oyster, Flat oyster |
|  |  |  | **Scallops** | Scallops, Giant Atlantic scallop |
|  | **Cephalopod** | | **Octopus** | Common octopus |
|  |  |  | **Squid** | Inshore squid, European squid, Ommastrephidae squids, Cuttlefish |
|  | **Gastropod** | | **Abalone** | Abalone |
|  |  |  | **Conch** | Conch shells |

**Supplementary Table 2.** Nutrient content per 100g by phylum and species group

| Spees group | N | Iron  (mg) | | Zinc  (mg) | | Calcium  (mg) | | Potassium  (mg) | | Magnesium  (mg) | | Selenium (mcg) | | Vit. B12 (mcg) | | Vit. A  (mcg) | | Vit. D  (mg) | | Omega 3  (g) | |
| --- | --- | --- | --- | --- | --- | --- | --- | --- | --- | --- | --- | --- | --- | --- | --- | --- | --- | --- | --- | --- | --- |
|  |  | Mean | SD | Mean | SD | Mean | SD | Mean | SD | Mean |  | Mean | SD | Mean | SD | Mean | SD | Mean | SD | Mean | SD |
| Crab | 40 | 0.93^efg^ | 0.69 | 4.49^b^ | 1.35 | 112^cde^ | 48.8 | 274^f^ | 83.6 | 48.9^eg^ | 16.2 | 48.8^de^ | 10.6 | 7.65^e^ | 2.13 | 35.3_c_ | 23.3 | 0.00^f^ | 0.00 | 0.23^g^ | 0.06 |
| Crayfish | 12 | 1.29^defg^ | 0.92 | 1.51^bcd^ | 0.42 | 39.8^cdef^ | 17.6 | 283^def^ | 38.2 | 28.8^fgh^ | 2.24 | 35.6^efg^ | 6.68 | 1.94^ghi^ | 0.19 | 15.4^defgi^ | 0.67 | 0.00^ef^ | 0.00 | 0.16^g^ | 0.02 |
| Lobster | 24 | 0.88^efg^ | 0.75 | 2.66^bcd^ | 0.6 | 57.0^cdef^ | 15.5 | 328^bcdef^ | 73.3 | 37.3^efgh^ | 10.3 | 59.3^cd^ | 19.2 | 1.34^hi^ | 0.41 | 5.72^ghi^ | 2.04 | 0.00^f^ | 0.00 | 0.24^g^ | 0.18 |
| Shrimp  /Prawn | 76 | 1.35^def^ | 0.98 | 1.82^bcd^ | 0.65 | 114^cd^ | 161 | 270^f^ | 88.6 | 45.9^efg^ | 11.6 | 41.8^ef^ | 14.5 | 3.44^fg^ | 2.11 | 14.9^efg^ | 13.1 | 0.00^f^ | 0.00 | 0.18^g^ | 0.09 |
| Bass | 12 | 1.04^defg^ | 0.10 | 0.79^bcd^ | 0.11 | 25.2^f^ | 8.08 | 393^abcd^ | 55.1 | 32.7^efgh^ | 3.21 | 25.4^g^ | 3.77 | 3.93^fghi^ | 0.5 | 13.6^defghi^ | 5.92 | 3.72^d^ | 0.38 | 0.83^cde^ | 0.20 |
| Catfish | 64 | 0.76^fg^ | 0.71 | 1.27^cd^ | 2.19 | 25.1^f^ | 13 | 310^def^ | 51.4 | 25.8^h^ | 4.67 | 27.3^g^ | 14.8 | 2.63^ghi^ | 1.42 | 12.9^efg^ | 9.42 | 0.80^ef^ | 0.57 | 0.33^g^ | 0.29 |
| Cod | 48 | 0.20^g^ | 0.07 | 0.45^d^ | 0.07 | 16.2^f^ | 7.41 | 394^ab^ | 67.5 | 29.0^h^ | 4.09 | 30.8^g^ | 5.53 | 1.47^h^ | 0.48 | 1.89^i^ | 0.56 | 1.44^e^ | 0.77 | 0.26^g^ | 0.10 |
| Mackerel | 12 | 1.24^defg^ | 0.19 | 0.52^bcd^ | 0.09 | 38.4^def^ | 12 | 433^a^ | 67.1 | 35.2^efgh^ | 5.23 | 66.2^bc^ | 8.6 | 7.81^e^ | 0.74 | 4.8^fghi^ | 0.59 | 30.1^a^ | 3.75 | 1.56^b^ | 0.88 |
| Pike | 12 | 0.21^fg^ | 0.06 | 0.97^bcd^ | 0.1 | 32^ef^ | 3.51 | 405^abc^ | 63.3 | 29.2^fgh^ | 2.96 | 33.7^efg^ | 19.3 | 13.7^d^ | 1.22 | 12.7^efghi^ | 0.87 | 3.47^d^ | 0.31 | 0.21^g^ | 0.05 |
| Salmon | 41 | 0.45^g^ | 0.21 | 0.50^d^ | 0.11 | 14.4^f^ | 4.83 | 396^ab^ | 58 | 28^h^ | 2.96 | 25.9^g^ | 4.61 | 4.7^f^ | 0.98 | 11.1^efghi^ | 3.35 | 9.38^c^ | 3.6 | 2.61^a^ | 0.67 |
| Sole | 8 | 0.51^defg^ | 0.15 | 0.56^bcd^ | 0.09 | 44.3^cdef^ | 19.4 | 363^abcdef^ | 46.9 | 31.3^efgh^ | 3.52 | 27.6^fg^ | 3.77 | 1.75^fghi^ | 0.37 | 5.52^efghi^ | 0.49 | 0.83^ef^ | 0.07 | 0.26^fg^ | 0.06 |
| Tilapia | 24 | 1.02^defg^ | 1.01 | 1.85^bcd^ | 2.83 | 40.8^f^ | 45.5 | 321^cdef^ | 43.6 | 30.1^fh^ | 4.52 | 29.3^g^ | 7.07 | 1.31^hi^ | 0.21 | 2.63^hi^ | 3.61 | 21.0^b^ | 1.07 | 0.16^g^ | 0.10 |
| Trout | 28 | 0.46^fg^ | 0.17 | 0.67^d^ | 0.32 | 22.6^f^ | 8.21 | 410^a^ | 58.7 | 28.6^h^ | 3.39 | 20.3^g^ | 3.4 | 4.05^fg^ | 0.49 | 17.8^def^ | 1.9 | 9.00^c^ | 2.88 | 0.82^ce^ | 0.00 |
| Abalone | 3 | 3.34^bcde^ | 0.76 | 1.4^bcd^ | 0.34 | 42.8^cdef^ | 10.4 | 291^abcdefg^ | 69.3 | 74.5^bcde^ | 16.8 | 27.9^efg^ | 6.34 | 5.38^efghi^ | 0.95 | 13.6^cdefghi^ | 2.61 | 0.00^ef^ | 0.00 | 0.10^dfg^ | 0.00 |
| Conch | 9 | 4.58^bc^ | 1.27 | 7.22^b^ | 2.11 | 445^a^ | 130 | 348^abcdef^ | 98 | 377^a^ | 104 | NA | NA | NA | NA | 15.0^defghi^ | 0 | 0.00^ef^ | 0.00 | 0.12^g^ | 0.00 |
| Clams | 6 | 9.17^a^ | 2.03 | 1.69^bcd^ | 0.37 | 282^b^ | 68.9 | 256^efg^ | 54.6 | 104^b^ | 21.0 | 160^a^ | 34.1 | 46.7^a^ | 7.34 | 102^a^ | 17.6 | 0.00^f^ | 0.00 | 0.44^cdefg^ | 0.30 |
| Mussels | 27 | 5.84^b^ | 3.49 | 3.01^bcd^ | 1.58 | 75.8^cdef^ | 30.5 | 256^f^ | 85.8 | 73.9^bc^ | 23.1 | 67.6^bc^ | 13.2 | 15.1^d^ | 4.96 | 83.2^b^ | 16.2 | 0.00^f^ | 0.00 | 0.70^cdef^ | 0.29 |
| Oysters | 27 | 4.54^c^ | 1.88 | 22^a^ | 17.9 | 126^c^ | 56.1 | 182^g^ | 52.6 | 55.0^de^ | 34.8 | 45.6_ef_ | 18.6 | 21.8^c^ | 4.51 | 26.9cd | 15 | 0.61^ef^ | 0.49 | 0.74^cde^ | 0.47 |
| Scallops | 6 | 1.48^defg^ | 0.67 | 2.89^bcd^ | 0.9 | 91.4^cdef^ | 33.2 | 408^abcde^ | 129 | 60.6^cdefg^ | 17.8 | 28.7^efg^ | 12.5 | 4.49^efghi^ | 1.44 | 9.9^defghi^ | 8.8 | 0.00^ef^ | 0.00 | 0.33^defg^ | 0.22 |
| Octopus | 12 | 4.12^c^ | 1.44 | 6.03^bc^ | 2.33 | 57.4^cdef^ | 14.7 | 382^abcde^ | 96.1 | 96.2^b^ | 25.1 | 79.5^b^ | 21.2 | 26.6^c^ | 6.42 | 39^c^ | 8.49 | 0.25^ef^ | 0.38 | 0.36^dfg^ | 0.12 |
| Squid |  |  |  |  |  |  |  |  |  |  |  |  |  |  |  |  |  |  |  |  |  |

| Legend |
| --- |
| >20% DV / 100 g |
| 10-20% DV / 100 g |
| <10% DV /100 g |

Note. Means within rows that do not share a superscript letter are significantly different at the p<.05 level based on Tukey’s HSD post hoc tests.

##### **Supplementary Table 3.** Price data by species groupings

| Species Grouping | Globefish Species | Cost/kg (EU) | Cost/kg (USD) | Yield | Cost/ Kg/ Yield | (Cost/Kg/  Yield)^-1^ | Price Index NRF6.2 | Price Index NRF9.2b |
| --- | --- | --- | --- | --- | --- | --- | --- | --- |
| Bass | (whole) European seabass | 6.18 | 6.74 | 0.39 | 17.28 | 0.06 | 6.13 | 8.10 |
| Catfish | (whole) Wolffishes or Atlantic catfish | 5.96 | 6.50 | 0.19 | 34.19 | 0.03 | 2.42 | 3.10 |
| Clams | (whole) Donax clams, imperial surf clam | 13.50 | 14.72 | 0.20 | 73.58 | 0.01 | 2.75 | 3.78 |
| Cod | (whole) Atlantic cod | 6.91 | 7.53 | 0.38 | 19.82 | 0.050 | 3.50 | 7.72 |
| Conch | (whole) Conch | 6.51 | 7.09 | 0.68 | 10.48 | 0.10 | 7.24 | 15.74 |
| Crab | (whole) Crab, edible crab, spinous spider crab, velvet swim crab | 12.23 | 13.33 | 0.25 | 53.32 | 0.02 | 3.00 | 3.43 |
| Lobster | (whole) Common spiny lobster, European lobster, Norway lobster | 31.60 | 34.44 | 0.28 | 123.01 | 0.01 | 0.70 | 1.41 |
| Mackerel | (whole) Atlantic horse mackerel | 4.07 | 4.44 | 0.54 | 8.22 | 0.12 | 12.54 | 35.18 |
| Mussels | (whole) Mediterranean mussel | 3.22 | 3.51 | 0.51 | 6.88 | 0.15 | 24.70 | 33.13 |
| Octopus | (whole) Common octopus | 12.03 | 13.11 | 0.79 | 16.60 | 0.06 | 9.46 | 12.47 |
| Oysters | (whole) Oysters | 16.47 | 17.95 | 0.11 | 163.15 | 0.01 | 1.40 | 1.49 |
| Pike | (whole) Pike perch | 9.00 | 9.81 | 0.38 | 25.82 | 0.04 | 4.46 | 6.35 |
| Salmon | (whole) Atlantic salmon | 7.85 | 8.56 | 0.65 | 13.16 | 0.08 | 6.38 | 12.31 |
| Scallops | (whole) Great Atlantic scallop | 4.87 | 5.30 | 0.13 | 40.79 | 0.03 | 3.11 | 3.16 |
| Shrimp/Prawn | (whole) Common prawn, giant tiger prawn, common shrimp, scarlet shrimp | 25.65 | 27.96 | 0.46 | 60.78 | 0.02 | 1.88 | 2.57 |
| Sole | (whole) Common sole, wedge sole, lemon sole | 18.08 | 19.71 | 0.60 | 32.85 | 0.03 | 2.51 | 4.23 |
| Squid | (whole) European squid, Indian squid, longfin squid, opalescent inshore squid, patagonian squid, squid | 6.77 | 7.38 | 0.78 | 9.46 | 0.11 | 12.58 | 21.99 |
| Tilapia | (fillet) Nile tilapia | 4.68 | 5.10 | 1.00 | 5.10 | 0.20 | 14.79 | 42.90 |
| Trout | (whole) Rainbow trout | 6.60 | 7.19 | 0.66 | 10.9 | 0.09 | 9.17 | 12.48 |
| Abalone | No data available | N/A | N/A | N/A | N/A | N/A | N/A | N/A |
| Crayfish | No data available | N/A | N/A | N/A | N/A | N/A | N/A | N/A |
